# Predicting *BRAF* Mutations in Cutaneous Melanoma Patients Using Neural Network Analysis

**DOI:** 10.1155/jskc/3690228

**Published:** 2024-12-19

**Authors:** Oleksandr Dudin, Ozar Mintser, Vitalii Gurianov, Nazarii Kobyliak, Dmytro Kaminskyi, Alina Matvieieva, Roman Shabalkov, Artem Mashukov, Oksana Sulaieva

**Affiliations:** ^1^Scientific Department, Medical Laboratory CSD, Kyiv, Ukraine; ^2^Department of Informatics, Information Technology and Transdisciplinary Learning, Shupyk National Healthcare University of Ukraine, Kyiv, Ukraine; ^3^Department of Health Care Management, Bogomolets National Medical University, Kyiv, Ukraine; ^4^Endocrinology Department, Bogomolets National Medical University, Kyiv, Ukraine; ^5^Department of Oncology and Radiotherapy, International Humanitarian University, Odesa, Ukraine

**Keywords:** BRAF mutation, cutaneous melanoma, multilayered perceptron, predictive model

## Abstract

Point mutations at codon 600 of the BRAF oncogene are the most common alterations in cutaneous melanoma (CM). Assessment of BRAF status allows to personalize patient management, though the affordability of molecular testing is limited in some countries. This study aimed to develop a model for predicting alteration in BRAF based on routinely available clinical and histological data.

**Methods:** For identifying the key factors associated with point mutations in BRAF, 2041 patients with CM were recruited in the study. The presence of BRAF mutations was an endpoint. The variables included demographic data (gender and age), anatomic location, stage, histological subtype, number of mitosis, and also such features as ulceration, Clark level, Breslow thickness, infiltration by lymphocytes, invasiveness, regression, microsatellites, and association with nevi.

**Results:** A relatively high rate of BRAF mutation was revealed in the Ukrainian cohort of patients with CM. BRAF-mutant melanoma was associated with younger age and location of nonsun-exposed skin. Besides, sex-specific differences were found between CM of various anatomic distributions and the frequency of distinct BRAF mutation subtypes.

A minimal set of variables linked to BRAF mutations, defined by the genetic input selection algorithm, included patient age, primary tumor location, histological type, lymphovascular invasion, ulceration, and association with nevi. To encounter nonlinear links, neural network modeling was applied resulting in a multilayer perceptron (MLP) with one hidden layer. Its architecture included four neurons with a logistic activation function. The AUROCMLP6 of the MLP model comprised 0.79 (95% CІ: 0.74–0.84). Under the optimal threshold, the model demonstrated the following parameters: sensitivity: 89.4% (95% CІ: 84.5%–93.1%), specificity: 50.7% (95% CІ: 42.2%–59.1%), positive predictive value: 73.1% (95% CІ: 69.6%–76.3%), and negative predictive value: 76.0% (95% CІ: 67.6%–82.8%). The developed MLP model enables the prediction of the mutation in BRAF oncogene in CM, alleviating decisions on personalized management of patients with CM.

In conclusion, the developed MLP model, which relies on the assessment of 6 variables, can predict the *BRAF* mutation status in patients with CM, supporting decisions on patient management.

## 1. Introduction

Management of patients with cutaneous melanoma (CM) depends on assessing molecular biomarkers that enable personalization of patient treatment. The genetic landscape of melanoma is complex and variable depending on histological subtype, location, and individual characteristics, which affects heterogeneous treatment outcomes [1, 2]. *BRAF* mutations in codon 600, including *c.1799_1800delinsAA (p.Val600Glu-V600E), c.1798_1799delinsAA (p.Val600Lys*–*V600K), c.1799_1800delinsAC (p.Val600Asp*–*V600D),* and *c.1798_1799delinsAG (p.Val600Arg*–*V600R*), are considered to be the most common genetic alteration in CM [3], the prevalence of which reaches 58% in the Ukrainian population [4]. Besides other non-V600 *BRAF* alterations were found in CM, though their rate is extremely low which neglects the clinical significance. Previously, BRAF mutations were shown to be associated with younger age and melanoma location at sun-shielded skin sites. However, these factors are insufficient to predict the probability of a *BRAF* mutation in every patient for prognostic purposes and to define a group of patients who can benefit from the targeted therapy [5, 6]. The detection of *BRAF* mutations is mostly based on PCR, although other methodologies (including immunohistochemistry and NGS) are also helpful for identifying various genetic alterations for further clinical decisions. Despite the high sensitivity and specificity, the costs and affordability of molecular methods in developing countries can be limitations for economically disadvantaged groups.

Alternatively, the application of machine learning techniques can be used for predicting the *BRAF* mutation status in CM using clinical and pathological data [7]. Indeed, the role of artificial intelligence (AI) in pathology is growing progressively and relies on machine learning methods. Figueroa-Silva et al. used an ML-based approach and defined seven variables, including age, Breslow thickness, Breslow density, epidermal contour hyperplastic, nests, metastases, and mitotic rate, for predicting the *BRAF* status. The developed tool demonstrated an AUC of 0.878 and was considered useful [7]. However, some variables of the model are outside of standard protocols for CM reporting, and their assessment requires additional time and procedures, complicating pathologists' work [8, 9].

The goal of this study was to develop tools for predicting *BRAF* mutations using routine clinical and histological features.

## 2. Materials and Methods

The study protocol was approved by the institutional review board (IRB of Medical Laboratory CSD, protocol No. 4/2020 from 16.10.2020) and followed the principles outlined in the Declaration of Helsinki for all human or animal experimental investigations.

A total of 2041 CM cases were retrieved from the database collected from 2017 to 2023 from the database of the Medical Laboratory CSD. Only patients with CMs of primary tumors were enrolled in this cross-sectional study. The study included two stages. In the first stage, we assessed the rate of *BRAF* mutations and their relationship with basic clinicodemographic data. In the second stage, the factors predicting *BRAF* mutation status were assessed using statistical analysis.

The collected clinicodemographic data included the patient's gender, age at CM diagnosis, and anatomical location of the primary melanoma lesion. Relevant histological characteristics according to CAP protocols for CM, including CM site, histological type according to WHO classification. Pathological stage, Breslow thickness, and Clark level were considered in complex with other histological data including lesion ulceration, number of mitosis, and density of tumor-associated lymphocytes (TILs) were retrieved. All patients were tested for *BRAF* codon 600 mutations. Molecular testing was conducted on formalin-fixed paraffin-embedded blocks with verified tumor content according to the algorithms described before [2]. Ten 10-μm-thick sections were cut from each formalin-fixed paraffin-embedded block containing a representative tumor area (> 20% tumor cells, or > 200 cells in the sample, with necrosis area less than 20%). DNA was extracted using the ZYTOVISION VisionArray FFPE DNA Extraction kit in line with the instructions of the manufacturer. *BRAF* mutation detection was conducted using the qPCR system “Easy PGX ready BRAF” (Diatech Pharmacogenetics, Italy) based on a real-time polymerase chain reaction. The assay detected 5 types of *BRAF* mutations in codon 600: V600E (1799T > A), V600E (1799_1800TG > AA), V600K (1798_1799GT > AA), V600D (1799_1800TG > AT), and V600R (1798_1799GT > AG).

Statistical analysis was conducted using MedCalc statistical software Version 22.016 (MedCalc Software Ltd., Ostend, Belgium), GraphPad Prism (GraphPad Prism Version 10.0.3, San Diego, California, USA), and Statistica Neural Networks 4.0 C (StatSoft, Inc., 1998-1999). Descriptive statistics for continuous variables (such as age, mitotic rate, and Breslow thickness) are presented as the mean ± SD. Quantitative data were assessed as frequencies (%). The *χ*^2^ test or Fisher's exact test was used to compare frequencies. An unpaired *t*-test was used to compare continuous variables.

Logistic regression and neural network analysis were applied for data analysis. At the first stage of analysis, 15 variables were used, including gender, age, anatomical location of the primary lesion, stage, histological type, ulceration, Clark level, Breslow thickness, number of mitosis, infiltration by lymphocytes, lymphovascular invasion (LVI), perineural invasion (PNI), features of regression, microsatellites, and association with nevi. In the next step, the most informative features were selected for building a predictive model. The diagnostic performance of the models was assessed using receiver operating characteristic (ROC) curve analysis. The area under the ROC curve (AUROC) and its 95% confidence interval (CI) were calculated. A *p* value < 0.05 was considered to indicate statistical significance in all the tests.

The method of development and analysis of neural networks was applied to assess the effect of variables on the outcome. The outcome indicator was *BRAF* status (variable Y): in the case of *BRAF* wild-type melanoma, *Y* = 0. When a *BRAF* mutation was detected, *Y* = 1 indicated a negative outcome. When constructing and analyzing mathematical forecasting models, all patients were randomly (using a random number generator) divided into 3 sets: training (which was used to build the model and calculate weight coefficients of the neural network), test (which was used to prevent overtraining of the mathematical model), and verification (which was used to test the predictive ability of the mathematical model on new data for controlling model retraining) sets.

## 3. Results

### 3.1. Prevalence of *BRAF* Mutations in CM in the Ukrainian Population

Overall, *BRAF* status was assessed in CM samples of 2041 patients with CM. Among them 1235 patients had CMs harboring *BRAF* mutations (60.5%). The average age of the patients was 54.2 ± 0.31 years (95% CI: 53.6–54.8).

Among the observed cohort, there were 991 males and 1050 females. Male sex was associated with younger age (52.6 ± 10.4 vs. 54.6 ± 14.3 years for males and females, respectively; *p* < 0.001) but not with the rate of *BRAF* mutation (*p*=0.171).

There was a significant association between *BRAF* mutations and CM location at sun-shielded sites (*p* < 0.001), with the highest prevalence of mutations in lesions located in the truncus and neck.

Among the *BRAF* mutation subtypes, the V600E variant prevailed in 88.6% (1094 of 1235 *BRAF*m patients). Moreover, the V600K mutation was detected in 122 patients (9.9%), and the V600D/R variant was detected in 19 (1.5%) patients. Despite the lack of differences in the incidence of *BRAF* mutations between men and women in Ukraine, we found specific sex differences in the prevalence of the BRAF mutation subtypes. The incidence of V600K and V600D/R was almost twice as high in the CM of males as in that of females (*p* < 0.001).

Although there was no statistically significant association between the *BRAF* mutation subtype and CM location (*p*=0.06), the V600K variant had a greater cranial prevalence (in the face, scalp, and neck) than did the truncus variant but was rare in the limbs. The *BRAF* V600D/R subtype had the highest prevalence in patients with scalp-arising melanoma (3.8% vs. 1.5% on average), reflecting a greater association with hairy skin.

Thus, the Ukrainian population demonstrated a high rate of *BRAF* mutation in CM, which was associated with younger age and location at sun-shielded sites. This study also revealed sex-specific differences in CM anatomic distribution and *BRAF* mutation subtype incidence.

Although the analysis of clinicodemographic data revealed an association between *BRAF* mutation status and age and tumor location, these data were not sufficient for predicting CM harboring *BRAF* mutations.

### 3.2. Neural Network Model for Predicting *BRAF* Status

The logistic regression method for predicting *BRAF* mutation in melanoma samples revealed a relatively weak correlation (AUROC_log15_ = 0.69; 95% CІ: 0.63–0.75) between *BRAF* status and 15 variables, although the model was adequate (*χ*^2^ = 35.5 at 20 degrees of freedom, *p* < 0.001).

**Figure 1:**
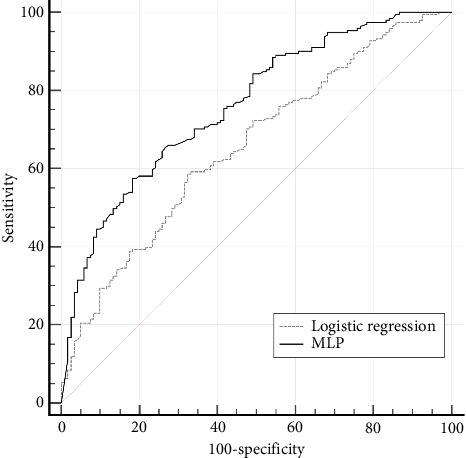
The performance characteristics of the 6-factorial MLP model for predicting *BRAF* mutations in CM samples compared to those of the logistic regression model. Characteristics of the MLP model compared to those of the logistic regression model with a difference between areas of Δ = 0.11 (95% CI: 0.04–0.17), *p*=0.001. The sensitivity of the MLP model was 89.4% (95% CІ: 84.5%–93.1%), the specificity was 50.7% (95% CІ: 42.2%–59.1%), the positive predictive value (PPV) was 73.1% (95% CІ: 69.6%–76.3%), and the NPV was 76.0% (95% CІ: 67.6%–82.8%).

By using the genetic selection method, the minimal set of variables related to *BRAF* mutations was defined and included 6 variables: age, primary tumor location, histological type, ulceration, LVI, and association with nevi (Table 1). These variables were used for building a 6-factorial logistic regression model that was not only adequate (*χ*^2^ = 25.1 at 11 degrees of freedom, *p*=0.009) but also demonstrated a weak predictive power, with AUROC_log6_ = 0.66 (95% CІ: 0.60–0.71) (Figure 1).

For encountering nonlinear links that cannot be considered in multiplicative (additive) models, the method of building nonlinear neural network models was applied. For this purpose, a multilayer perceptron (MLP) with one hidden layer was used. The hidden layer architecture included 4 neurons with a logistic activation function.

The AUROC_MLP6_ of the model was 0.79 (95% CІ: 0.74–0.84), which reflects the good consistency of the *BRAF* mutation risk prediction model using 6 variables, such as age, primary tumor location, histological type, ulceration, LVI, and association with a nevus (Figure 1).

The efficiency of the neural network model also reflects the significant nonlinear characteristics of BRAF status and can be used for predicting *BRAF* mutations in CM.

The critical threshold for this model is chosen based on the optimization of false positive and false negative predictions. When applying the optimal (by the Youden index) threshold, the following characteristics of the model were reached: sensitivity, 89.4% (95% CІ: 84.5%–93.1%); specificity, 50.7% (95% CІ: 42.2%–59.1%); positive predictive value (PPV), 73.1% (95% CІ: 69.6%–76.3%); and NPV, 76.0% (95% CІ: 67.6%–82.8%). For practical application as an algorithm for decision-making, the neural network model was realized in the Excel's Table (LibreOffice 24.8). The developed MLP model allows the prediction of the BRAF mutation status in CM, facilitating decisions concerning further patient management.

## 4. Discussion

This study demonstrated a relatively high rate of *BRAF* mutation in Ukrainian patients with CM, as compared to other populations demonstrating *BRAF* mutation prevalence of 36%–50% [10]. Mutations in BRAF were associated with younger age and location on sun-shielded skin. Although many authors have previously demonstrated differences in the *BRAF* mutation rate between males and females, we did not find a link between this genetic alteration and sex [11]. At the same time, in the Ukrainian cohort, there were sex differences in the incidence of V600K (12.8 vs. 7.2%) and V600D/R (2.2% vs. 0.9%), which was almost twice as high in the CM of males as in that of females (*p* < 0.001). These data correspond with those of Van der Kooij and colleagues, who, in a nationwide cohort study of 38,985 CM patients, demonstrated a greater percentage of V600K mutations in men (8.8%) than in women (4.2%) [11]. At the same time, some other studies did not reveal sex differences in various subtypes of *BRAF* mutations, but they were conducted on much smaller samples [3, 12].

Notably, the V600K mutation, which is associated with greater activation of the PIK3CA pathway and more aggressive CM behavior [5, 13], was detected in almost 10% of CM patients with *BRAF* mutations in Ukraine, which aligns with global statistics [14]. Despite the close association between *BRAF* mutations and the truncal location of primary tumors revealed in the present study, the V600K mutation had a cranial prevalence, representing 23.8% of all *BRAF*-mutated CMs on the face and 19.2% of *BRAF* mutation cases in the neck. Similar data were reported by Menzies et al. [15]. The *BRAF* V600D/R subtype had the highest prevalence in patients with scalp-arising melanoma (3.8% vs. 1.5%), reflecting a greater association with hairy skin [15]. Knowing the rate of V600K and V600D/R mutations is essential for planning immunotherapy and developing alternative treatment options for CM.

In the second stage of this study, we developed a model for predicting *BRAF*, using logistic regression and neural network modeling. Other models were shown to be effective in predicting BRAF mutations in CM. For instance, a retrospective observational study based on 106 cases of invasive melanoma analysis of clinical and histologic variables applied a machine learning approach. The authors used SHapley Additive exPlanations (SHAP) to define a heuristic model for evaluating BRAF mutation probability. Age, Breslow thickness, and Breslow density were defined as the most significant variables for predicting BRAF mutation probability [7]. Besides, three different models, including a binary logistic regression model, a classification and regression analysis model, and a random forest model, were used for forecasting the probability of BRAF mutation in CM [16]. All three models demonstrated the significance of age, histological type, and location of the primary tumor that were also confirmed in the current study [16]. Finally, Schneider et al. used a multimodal classifier relying on machine learning algorithms for predicting BRAF mutation presence in primary and metastatic melanomas and demonstrated higher performance when combining clinical, histological, and epigenetic data [17]. In our study, 6 main variables were defined as factors nonlinearly related to *BRAF* mutations with no respect to *BRAF* mutations' subtype. These variables included age, primary tumor location, histological type, ulceration, LVI, and association with a nevus.

This study revealed the predictive value of age and primary tumor site of the trunk, which aligns with the previously reported association of *BRAF*-mutated CM with younger age and location at the site with little or moderate sun-induced damage, including to the trunk [4, 18–20]. Early onset, anatomic site, and lack of relation to UV damage in *BRAF*-mutated CM reflect the distinct pathogenic pathways of melanocyte malignization, which are different from those of wild-type melanoma [18] and can also define the roots of the found predictive significance of the associated nevus on the probability of harboring *BRAF* mutations in CM.

Indeed, the acquisition of a *BRAF* mutation was suggested to be an initiating event in melanocytic neoplasia, including both nevi and melanoma [21]. In the Takata study, *BRAF* mutations were found not only in CMs but also in contiguous nevi, so the authors suggested that oncogenic *BRAF* mutations could contribute to benign melanocytic proliferation with further switching to invasive melanoma [22]. In fact, *BRAF* controls many aspects of stepwise melanomogenesis and can be an early event in CM evolution, provoking genomic instability and the acquisition of a wide spectrum of new genetic alterations [23, 24]. On the other hand, *BRAF* mutations are detected in approximately 80% of nevi, which could undermine the role of *BRAF* alterations in melanoma development and progression. This paradox has been revealed by *in vitro* studies demonstrating that overexpression of the RAS–RAF–mitogen-activated protein kinase (MAPK) pathway in *BRAF*-mutated melanocytic lesions promotes not only increased melanocyte proliferation but also rapid melanocyte senescence [25]. This effect is a protective mechanism known as oncogene-induced senescence [26] and is related to the preservation of tumor suppressors, including *TP53* and *PTEN*, which results in melanocyte senescence and cell cycle arrest by activating p15^INK4b^, p16^INK4a^, p19, and acidic *β*-galactosidase [5]. Although *TP53* alterations are rare in CM, the downregulation of *PTEN* combined with activation of the PI3K/Akt signaling pathway is quite common in nevi. This mechanism is considered to be responsible for abolishing senescence and allowing further progression to dysplasia and transformation to melanoma [27–29]. Therefore, as an initial genetic alteration in nevus-to-melanoma evolution, *BRAF* mutation can be predicted by the association of CM with a nevus, especially in patients with CM located at the trunk (nonsun-exposed skin).

The presence of superficial spreading histology was also shown to predict *BRAF* mutations in CM. This finding aligns with the known association of *BRAF* mutation with particular forms of CM, and approximately 80% of *BRAF*-mutated melanomas are superficial or nodular [30] melanomas. Nevertheless, in our model, a strong ability to predict *BRAF* status was revealed for superficially spreading melanoma, although the relation to ulceration was also significant. Importantly, the model also revealed that LVI was related to the probability of *BRAF* mutation. The oncogenic *BRAF* cascade involves many signaling pathways in melanocytes, including the *MITF* signaling pathway. Mutated *BRAF* exerts exquisite control over *MITF*, which regulates the expression of key cell cycle facilitators, such as *CDK2* and *CDK4*, stimulating melanoma cell proliferation, survival, and phenotype switching from proliferative and invasive states [5, 31]. In addition, activation of the MAPK pathway in *BRAF*-mutated CM can also be associated with increased production of VEGF, provoking LVI.

### 4.1. Limitations of the Study

This was a retrospective study assessing only a subset of *BRAF V600* variants rate in CM. No other genetic alterations were considered in this study. Although the developed model demonstrated a relatively high performance, further research is needed for its validation in different populations.

## 5. Conclusion

In conclusion, the developed MLP model, which relies on the analysis of 6 variables, can predict the BRAF mutation status in CM patients, facilitating decisions concerning further patient management.

## Figures and Tables

**Table 1:** Characteristics of the 6-factorial logistic regression model for predicting *BRAF* mutation in CM samples.

Variables	Model coefficient, *b* ± *m*	*p*	OR (95% CI)
Age, per 1 year	−0.015 + 0.009	0.086	0.98 (0.97–1.00)
Primary tumor location			
Trunk	Referent		
Face	−1.58 ± 0.80	0.047	0.21 (0.04–0.98)
Limbs	−0.84 ± 0.33	0.010	0.43 (0.23–0.82)
NOS	−0.60 ± 0.32	0.059	0.55 (0.29–1.02)
Scapl	−1.26 ± 0.59	0.034	0.28 (0.09–0.91)
Histological type			
NOS	Referent		
NM	0.59 ± 0.45	0.189	1.81 (0.75–4.38)
Spitz	−0.45 ± 0.69	0.511	0.63 (0.16–2.46)
SSM	0.62 ± 0.28	0.025	1.85 (1.08–3.18)
Ulceration	0.20 ± 0.26	0.431	1.23 (0.74–2.04)
LVI	0.25 ± 0.33	0.597	1.29 (0.68–2.45)
Association with a nevus	0.33 ± 0.43	0.447	1.39 (0.60–3.24)

Abbreviations: LVI, lymphovascular invasion; NM, nodular melanoma; NOS, not otherwise specified; SSM, superficially spreading melanoma.

## Data Availability

The data will be made available upon request to the corresponding authors.
